# Toxicity and Pharmacokinetic Studies of Lidocaine and Its Active Metabolite, Monoethylglycinexylidide, in Goat Kids

**DOI:** 10.3390/ani8080142

**Published:** 2018-08-20

**Authors:** Dinakaran Venkatachalam, Paul Chambers, Kavitha Kongara, Preet Singh

**Affiliations:** School of Veterinary Science, Massey University, Tennent Drive, Palmerston North 4442, New Zealand; J.P.Chambers@massey.ac.nz (P.C.); K.Kongara@massey.ac.nz (K.K.); P.M.Singh@massey.ac.nz (P.S.)

**Keywords:** disbudding, goat kids, lidocaine hydrochloride, toxicity, pharmacokinetics

## Abstract

**Simple Summary:**

Disbudding is becoming a routine husbandry procedure in goat farms even though it is a painful procedure without appropriate pain relief. One of the ways to alleviate or minimize the pain associated with disbudding is by using local anesthetics like lidocaine hydrochloride. However, lidocaine hydrochloride has been reported to be toxic in goat kids and there is some data regarding the doses that produce toxicity in goat kids. Therefore, the research team studied the toxicity and pharmacokinetics of lidocaine hydrochloride in goat kids to recommend a safe dose for disbudding.

**Abstract:**

This study determined the convulsant plasma concentrations and pharmacokinetic parameters following cornual nerve block and compared the results to recommend a safe dose of lidocaine hydrochloride for goat kids. The plasma concentrations of lidocaine and monoethylglycinexylidide (MGX) were quantified using liquid chromatography-mass spectrometry. A total dose of 7 mg/kg body weight (BW) was tolerated and should therefore be safe for local and regional anesthesia in goat kids. The mean plasma concentration and mean total dose that produced convulsions in goat kids were 13.59 ± 2.34 µg/mL and 12.31 ± 1.42 mg/kg BW (mean ± S.D.), respectively. The absorption of lidocaine following subcutaneous administration was rapid with C_max_ and T_max_ of 2.12 ± 0.81 µg/mL and 0.33 ± 0.11 h, respectively. The elimination half-lives (t_½λz_) of lidocaine hydrochloride and MGX were 1.71 ± 0.51 h and 3.19 ± 1.21 h, respectively. Injection of 1% lidocaine hydrochloride (0.5 mL/site) was safe and effective in blocking the nerves supplying horn buds in goat kids.

## 1. Introduction

Disbudding is performed in domestic ruminants to prevent injuries among handlers or herd mates, to avoid damage to farm facilities and to facilitate the use of head bails [1,2,3,4]. Additionally, hornless animals require less feeding trough space and are easier to handle and transport than horned animals [2,5]. Disbudding in dairy goats is becoming a routine husbandry procedure even though it is a very stressful and painful procedure when performed without appropriate pain relief [6,7]. Selective breeding for polledness can eliminate the need for disbudding, but in certain breeds of goats (Saanen, Alpine and Toggenburg), the polled condition is associated with serious reproductive disorders in both sexes. Therefore, disbudding is inevitable in such goat breeds [8]. Thermal cauterization is the most commonly used technique but it is painful and stressful without appropriate anesthesia and analgesia [8,9]. Therefore, it is recommended to provide pain relief to improve the welfare of the animals undergoing disbudding [3,6].

Effective local anesthesia is one of the ways to alleviate or minimize the pain associated with disbudding [6,7]. Lidocaine is the commonly used local anesthetics in veterinary medicine but it has a historical reputation of being toxic to goat kids [8,10]. The reasons for lidocaine toxicity are associated with overdosing of the goat kids by not considering their body weight and the increased chances of systemic absorption of the drug from the highly vascularized injection sites [11]. The requirement for injecting local anesthetics at two nerve sites per horn bud (only one site in cattle) for cornual nerve block increases the total dose, which increases the chances of toxicity in goat kids as verses calves. Even though goat kids have been reported to be sensitive to lidocaine, there are no data on the plasma concentrations of lidocaine that caused toxicity [8,10]. To determine the toxic dose of the local anesthetics, it is important to determine its toxic plasma/serum concentrations [11]. Therefore, the objectives of this study are to determine the toxic dose, its corresponding plasma concentrations, and compare the results with pharmacokinetic parameters following cornual nerve block in goat kids to recommend a safe dose for cornual nerve block.

## 2. Materials and Methods

### 2.1. Reagents and Drugs

Reference standards of lidocaine hydrochloride and monoethylglycinexylidide (≥95%) were purchased from Sigma Aldrich, Auckland, New Zealand. Lidocaine hydrochloride for injection was purchased from Ethical agents Ltd., Auckland, New Zealand. Acetonitrile, methanol, water, and formic acid were mass spectrometry grade and were purchased from Fisher Scientific, Auckland, New Zealand. Heparin sodium and normal saline were purchased from Pfizer New Zealand Limited, Auckland, New Zealand, and Baxter Healthcare Pty Ltd., Old Toongabbie, NSW, Australia, respectively. Artificial colostrum and milk replacer were purchased from Farmlands Co-Operative Society Ltd., Palmerston North, New Zealand.

### 2.2. Experimental Animals

The study was conducted on healthy male Saanen goat kids collected from a commercial dairy goat farm. Kids were separated from their dams after receiving colostrum and were transported to the Massey University research facility. Animals were housed in pens with clean, dry straw bedding and heating lamps to keep the pens warm. All animals received artificial colostrum on the day of arrival using feeding bottles and then fed with milk replacers via milk feeding buckets. Milk feeding buckets remained in the pens for ad libitum access to milk replacer. The study procedures were approved by the Massey University Animal Ethics Committee (Protocol number-MUAEC Protocol 17/41 and 17/54).

### 2.3. Study Design

#### 2.3.1. Dose-Ranging Study

The aim of this experiment was to determine the maximum dose that can be safely used in goat kids (by any parenteral route) without any adverse effects. This experiment was conducted on three male Saanen goat kids (7–10 days old, weighing 6.4 kg to 6.9 kg). Both right and left cephalic veins were catheterized using 20 gauge, 48 mm intravenous (I/V) catheter (BD Insyte, Sandy, UT, USA), for administration of 2% lidocaine hydrochloride and pentobarbitone sodium in the contralateral vein. Three doses (7 mg/kg body weight (BW), 9 mg/kg BW, and 10 mg/kg BW) of lidocaine hydrochloride were tested (one animal/dose) by intravenous infusion over 60 s using a syringe pump (World Precision Instruments, Sarasota, FL, USA) and observed for toxicity signs (sedation and convulsions). Infusion was stopped when convulsions appeared and animals were immediately euthanized by intravenous injection of pentobarbitone sodium (100 mg/kg BW).

#### 2.3.2. Determination of Toxic Dose of Lidocaine and Its Corresponding Plasma Concentrations

Six male Saanen goat kids (seven to 10 days old, weighing 6.1 to 7.5 kg BW) were used to determine the convulsive dose and its corresponding plasma concentrations. The left cephalic vein was catheterized (20 gauge, 48 mm I/V catheter (BD Insyte, Sandy, UT, USA)) for intravenous infusion of 2% lidocaine hydrochloride (2 mg/kg/min) using a syringe pump and the right cephalic vein was catheterized for the collection of blood samples. Blood samples were collected prior to drug administration at 1-min intervals and at the time of onset of convulsions. The total dose required to produce convulsions was calculated using the data from the infusion pump and the plasma concentrations of lidocaine and its metabolite, monoethylglycinexylidide (MGX), in the collected samples were analyzed using a sensitive and simple liquid chromatography-mass spectrometry (LC-MS/MS) method described later. Drug administration was terminated when convulsions were observed and animals were euthanized immediately by intravenous injection of pentobarbitone sodium (100 mg/kg BW).

#### 2.3.3. Pharmacokinetics of Lidocaine and Its Metabolite, MGX, Following Cornual Nerve Block in Goat Kids

This experiment included 10 male Saanen goat kids less than a week old with a body weight range of 3.3 kg to 4.7 kg. Animals were restrained gently and 1% lidocaine hydrochloride (diluted in normal saline) was injected (0.5 mL per site) subcutaneously within 2 min around the cornual branches of the lacrimal and infratrochlear nerves of both the horn buds based on the procedure described by Sherman and Smith, 2009. The syringe plunger was pulled back prior to injection to ensure the needle was not in a blood vessel. After confirming the effect of the nerve blockade by pricking with a needle, horn buds were disbudded using a gas dehorner (Portasol, Elmira, OR, USA). Although this is not a proper behavioral study, animals were still monitored for pain-associated behaviors such as head scratching, head shaking, vocalization, body shaking, and toxicity signs for 3 h after drug administration. Blood samples (1 mL) were collected via the catheter in the cephalic vein prior to drug administration (0 min) and at 10 min, 20 min, 30 min, and 40 min and 1 h, 2 h, 4 h, 6 h, 8 h, and 12 h following drug administration. Immediately after collection, blood samples were cooled on ice and plasma was separated and stored at −20 °C until analysis.

### 2.4. Analytical Procedure

#### 2.4.1. Liquid Chromatography-Mass Spectrometry

A sensitive and a simple LC-MS/MS method using Parallel reaction monitoring (PRM) mode was developed and validated to quantify the plasma concentrations of lidocaine and MGX.

##### Instrumentation and Conditions

The Ultra High Performance Liquid Chromatography system (Thermo Scientific™ Dionex UltiMate™ 3000 System, Germering, Germany) was equipped with a quaternary pump (Dionex Ultimate 3000 RS pump), a vacuum degasser, a column compartment (Dionex Ultimate 3000 RS Column Compartment), and an auto-sampler (Dionex Ultimate 3000 RS Autosampler). The analytes were separated using a 2.6 µm particle size C-18 column (Accucore 100 mm × 2.1 mm, Auckland, New Zealand) coupled with a security guard column (Accucore Defender Guard Column, Auckland, New Zealand) maintained at a temperature of 25 °C. The mobile phase consisted of 0.1% formic acid and acetonitrile (70:30, *V*/*V*) and was delivered at a flow rate of 0.3 mL/minute. The PRM analyses were carried out on a hybrid quadrupole orbitrap mass spectrometer (Q Exactive™ Focus Hybrid Quadrupole-Orbitrap™ Mass Spectrometer, Thermo Scientific™, Bremen, Germany) with an electrospray-ionization interface. The precursor ions of lidocaine (*m*/*z* 235.180) and MGX (*m*/*z* 207.148) were included in the target list and were fragmented into their respective daughter ions using collision energy of 35 eV, which were detected using a resolution of 35,000 FWHM. Data processing was performed using the Thermo Scientific™ Xcalibur^®^ data system and quantitation was performed using peak-area ratios of the daughter ions of lidocaine (*m*/*z* 86.096) and MGX (*m*/*z* 58.065). Samples that exceeded the calibration limit were appropriately diluted with blank drug-free plasma and re-analyzed.

#### 2.4.2. Sample Preparation

An aliquot of 240 µL plasma was taken in a 1.5 mL Eppendorf centrifuge tube and mixed with 480 µL of ice-cold methanol and vortexed for 10 s. After 10 min, the samples were vortexed again and centrifuged at 14,000 rpm for 10 min. The clear supernatant (200 µL) was mixed with 0.1% Formic acid (200 µL) and centrifuged at 14,000 rpm for 10 min. Then the supernatant was taken into the autosampler vials and 10 µL was injected into the column.

#### 2.4.3. Preparation of Standards and Quality Control Samples

Standard stock solutions (1 mg/mL) of lidocaine hydrochloride and MGX were prepared by dissolving in methanol. Equal volumes of both the standard solutions were mixed and working solutions were then serially diluted using methanol. Calibration standards and quality control samples (0.0125 µg/mL, 0.125 µg/mL, and 1.250 µg/mL) were prepared freshly by spiking ice cold pooled blank goat plasma with working solutions.

#### 2.4.4. LC-MS/MS Method Validation

Specificity of the method was determined by analyzing blank goat plasma samples and samples spiked with lidocaine hydrochloride and MGX. The linearity of the method was determined by linear regression analysis calculated using the least square regression method. Calibration curves (0.00125 µg/mL to 2.50 µg/mL) were built using pooled plasma obtained from untreated goat kids. The lower limit of detection and quantification of the compounds were determined by signal-to-noise ratios of 3:1 and 10:1, respectively. Recoveries (0.0125 µg/mL, 0.125 µg/mL, and 1.250 µg/mL) from goat plasma were calculated by comparing the peak areas of spiked samples with control standards following the same sample preparation procedure described above. Intraday and interday precision and accuracy of the method were determined by running different concentrations of an independently prepared spiked goat plasma sample on the same day and for six different days, respectively. Carryover from the system was assessed by injecting blank plasma sample after an injection of the spiked plasma sample containing 2.50 µg/mL of compounds.

### 2.5. Pharmacokinetic Analysis

Pharmacokinetic parameters following subcutaneous injection were determined using noncompartmental analysis. PKSolver ‘add-on’ for Excel 2010 was used to calculate pharmacokinetic parameters using individual plasma concentration data [12]. The maximum plasma concentration (C_max_) and time to achieve C_max_ (T_max_) were determined directly from the plasma concentration data. The rate constant of the terminal phase (λ_z_) was calculated by linear regression of the logarithmic plasma concentration. Half-life of the terminal phase (t_½λz_) was calculated as ln_2_/λ_z_. The area under the curve (AUC) and the area under the first moment (AUMC) were determined using the linear trapezoidal method. Mean residence time (MRT) was calculated as AUMC/AUC. Data are reported in mean ± S.D.

## 3. Results

### 3.1. LC-MS/MS Method Validation

Representative chromatograms and mass spectra (obtained in PRM mode) of blank goat plasma, blank plasma spiked with 0.00125 µg/mL of analytes, and the plasma sample from an experimental animal are shown in Supplementary Material Figure S1a–c, respectively. The retention times of lidocaine and MGX were 0.97 min and 0.93 min, respectively, and the total run time was 4 min. The calibration curves (Supplementary Material Figure S2a,b) were linear over the concentration range of 0.00125 µg/mL to 2.50 µg/mL with a correlation coefficient (r^2^) of 0.9972 for lidocaine and with r^2^ of 0.9997 for MGX. The lower limit of quantification and detection for both lidocaine and MGX were 0.00125 µg/mL and 0.0005 µg/mL, respectively. The relative standard deviation of intraday assay for lidocaine and MGX were ≤5.7% and ≤5.3%, respectively. The relative standard deviation for interday assay for lidocaine and MGX were ≤12.69% and ≤15.64%, respectively. The recoveries for lidocaine ranged from 78% to 84% and for MGX were from 61% to 80%. No carry-over effect was found after the injection of spiked samples containing the upper limit of quantification.

### 3.2. Animal Experiments

All animals were healthy prior to drug administration. In the dose-ranging experiment, no adverse effects were noted after the infusion of 7 mg/kg BW over 60 s but, in the animals that received 9 mg/kg and 10 mg/kg, sedation followed by ataxia and convulsions were observed even before the completion of the infusion. Individual animal doses and plasma concentrations of lidocaine and its metabolite, MGX required to produce convulsions in goat kids after intravenous infusion of lidocaine hydrochloride were presented in Table 1. The average dosage required to produce convulsions in goat kids was 12.31 ± 1.42 mg/kg and the average plasma concentrations of lidocaine and MGX required to produce was 13.59 µg/mL and 0.39 µg/mL, respectively. All the animals had identical sequence of toxicity symptoms like sedation followed by ataxia and tonic-clonic convulsions.

**Figure 1:**
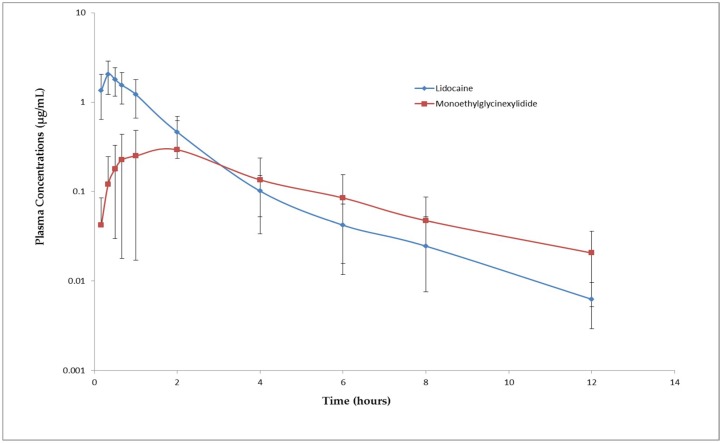
Mean (±S.D.) plasma concentrations of lidocaine (Red line) and its metabolite, monoethylglycinexylidide (blue line), in goat kids following cornual nerve block using 1% lidocaine (0.5 mL per site). Note the log scale of the *y*-axis.

The mean pharmacokinetic parameters of lidocaine and its metabolite are presented in Table 2. The plasma concentrations of lidocaine and MGX vs. time following subcutaneous administration are shown in Figure 1. The plasma concentrations of lidocaine and MGX were below the limit of detection in the samples collected prior to drug administration and were above the limit of quantification for up to 12 h post drug administration. The mean peak plasma concentration 2.12 ± 0.81 µg/mL (range 1.32–4.05 µg/mL) of lidocaine was reached at around 0.33 ± 0.11 h. The C_max_ and T_max_ of MGX were 0.31 ± 0.32 µg/mL and 1.53 ± 0.61 h, respectively. The elimination half-lives of lidocaine and MGX were 1.71 ± 0.51 h and 3.22 ± 1.21 h, respectively. Following subcutaneous administration of lidocaine (0.5 mL/site) at the nerve sites, the effective nerve blockade was produced and no pain-related signs were observed during disbudding but head scratching and head shaking were observed after 20 min. However, no lidocaine-related toxic signs were observed in any of the animals.

## 4. Discussion

A simple and sensitive LC-MS/MS method using PRM mode has been developed and validated for the simultaneous quantification of lidocaine and its metabolite MGX in goat plasma. The validation parameters were within the acceptable range.

Lidocaine hydrochloride has been widely used as a local anesthetic in both veterinary and human medicine but toxicity can occur if excessive drug doses are administered or because of accidental intravenous injection [13]. In goats, lidocaine has been reported to have historical reputation of being toxic especially during cornual nerve block in young animals [8,11]. However, there is little data on the toxic dose and the plasma concentration at which toxicity was observed. Only one study reported that convulsions were observed in a goat kid following intramuscular injection of lidocaine at approximately 10 mg/kg but there is no report about the plasma concentration at which toxicity occurred [10]. The toxic concentrations and pharmacokinetics of lidocaine have been studied in humans, sheep, dogs, and horses, but, to our knowledge, there are no reports in goats [14,15,16,17]. This is the first study that reports the pharmacokinetics and convulsive toxic doses and its concentrations in goat kids.

In the dose-ranging study, a dose of 7 mg/kg BW administered intravenously over a period of 60 s did not produce any observable toxicity signs. Therefore, this dose should be safe to use in goat kids for cornual nerve block since toxicity is unlikely to occur even if this dose is accidentally injected into veins. In addition, a total dose of 7 mg/kg BW may be considered safe for other local and regional nerve blocks in goat kids even though safety studies using higher numbers of animals are required before using this dose clinically.

The mean toxic dose required to produce convulsions in the goat kids (12.42 mg/kg) is less than that reported in newborn sheep (18.40 mg/kg) [15]. The mean plasma concentration required to produce convulsions in the present study is (13.59 ± 2.34 µg/mL) similar to that reported in newborn sheep (16.6 ± 1.2 µg/mL) [15]. In dogs, the mean concentration of lidocaine that produced toxicity was 8.21 + 1.69 µg/mL, which is lower than that found in the present study (13.59 ± 2.34 µg/mL) [16]. Meyer et al. (2001) found that, in horses, the serum concentration (3.24 ± 0.74 μg/mL) that produced intoxication is significantly lower than that found in goat kids [17]. The differences could be because of the different end points used to determine the toxicity in various species. In dogs, the tonic extension phase was considered as the toxic sign while, in horses, skeletal muscle fasciculation was used [16,17]. In the present study, convulsions were used as the end point. Another possible reason for the differences could be that the rate of drug administration used in different species was different [16,17]. Species and age differences have been reported to occur in the toxicity of lidocaine [15,16,17]. Young animals are less sensitive to lidocaine toxicity than adult animals because of higher volume of distribution in young animals [11,12,13,14,15]. The limitation of our study is that cardiovascular parameters were not recorded. However, it should be noted that the central nervous system is more sensitive than the cardiovascular system. Two to four times higher concentrations are required to produce cardiovascular toxicity signs than central nervous system signs [11,12,13,14,15].

The absorption of lidocaine following subcutaneous administration was rapid with an average T_max_ of 0.33 ± 0.11 h. The rate of elimination of lidocaine and MGX was moderate with a mean t_½λz_ of 2.28 h and 3.20 h, respectively. The mean peak plasma concentration of lidocaine (2.12 ± 0.81 µg/mL) observed after subcutaneous administration is almost 6.5 times less than the mean plasma concentration that produced convulsions (13.59 ± 2.34 µg/mL). The toxic dose of local anesthetics depends on the peak plasma concentration of the drug. Lower peak plasma concentrations reduce the chances of developing toxicity [18]. Since the C_max_ following injection of 0.5 mL/site of 1% lidocaine hydrochloride is significantly lower than the toxic plasma concentration. This dose may be safe for cornual nerve block in goat kids. The dose used in this study is not only safe but also effective in blocking the nerves supplying horn buds since no pain related to behavioral signs were observed during disbudding. However, most of the goat kids started showing signs like head scratching and head shaking 20 min after drug administration, which means that the anesthetic effect lasted only for 20 min. Increasing the concentration of lidocaine may increase the duration of the anesthetic effect. However, studies have shown that administration of lidocaine alone is not sufficient to provide pain relief for disbudding [6].

## 5. Conclusions

Based on the results of the present study, 7 mg/kg bodyweight may be safe to use in goat kids for local and regional nerve blocks. Yet, further studies using more animals are warranted before clinical use of this dose. The toxic conclusive concentrations determined in this study may be used as a standard to compare the peak plasma concentrations following various routes of administration to determine the safe dose in goat kids. In addition, injection of 0.5 mL per site of 1% lidocaine is safe and effective to produce cornual nerve block in goat kids. Significant differences observed in the peak plasma concentration and the convulsant plasma concentration suggests that use of higher lidocaine hydrochloride concentration (1.5% to 2%) for cornual nerve block in goat kids could be safe and could increase the duration of anesthesia. However, further pharmacokinetic and efficacy studies to determine the peak plasma concentrations and duration of analgesia using those doses are required to assess the safety before clinical use.

## Figures and Tables

**Table 1:** Individual animal doses and plasma concentrations of lidocaine and its metabolite, monoethylglycinexylidide, which produced convulsions in goat kids following intravenous infusion of 2% lidocaine hydrochloride (2 mg/kg/min).

Animal Number	Total Dose (mg/kg)	Plasma Concentrations (µg/mL)	
		Lidocaine	Monoethylglycinexylidide
1	10.23	12.68	0.31
2	11.70	12.64	0.49
3	13.84	15.01	0.62
4	13.98	17.35	0.39
5	11.76	13.33	0.26
6	12.33	10.53	0.26
Mean ± S.D. (standard deviation)	12.31 ± 1.42	13.59 ± 2.34	0.39 ± 0.14

**Table 2:** Mean ± S.D. pharmacokinetic parameters of lidocaine and monoethylglycinexylidide following subcutaneous administration of 1% lidocaine hydrochloride (0.5 mL per site) around the cornual branches of the lacrimal and infratrochlear nerves of both the horn buds in goat kids (*n* = 10).

Parameter	Lidocaine	Monoethylglycinexylidide
C_max_^1^ (µg/mL)	2.12 ± 0.81	0.31 ± 0.32
T_max_^2^ (h)	0.33 ± 0.11	1.53 ± 0.61
AUC (µg/mL h) ^3^	3.12 ± 1.09	1.34 ± 1.20
Terminal half-life (h)	1.71 ± 0.51	3.22 ± 1.21
Mean residence time (h)	1.59 ± 0.36	4.67 ± 1.34

^1^ Maximum plasma concentration, ^2^ time to reach maximum plasma concentration, ^3^ and area under the curve.

## Supplementary Materials

The following are available online at http://www.mdpi.com/2076-2615/8/8/142/s1, Figure S1: Chromatograms and mass spectra (obtained in PRM mode) of (**a**) blank goat plasma, (**b**) blank plasma spiked with 0.00125 µg/mL of lidocaine and monoethylglycinexylidide, and (**c**) plasma from an experimental animal after cornual nerve block, Figure S2. Calibration curves (0.00125–2.50 µg/mL) of lidocaine (**a**,**b**) monoethylglycinexylidide constructed by spiking pooled plasma from untreated goat kids.
