# Supplementary material for: Toxicity and Pharmacokinetic Studies of Lidocaine and Its Active Metabolite, Monoethylglycinexylidide, in Goat Kids
Source: Animals (Basel). 2018 Aug 20;8(8):142. doi: 10.3390/ani8080142 (PMC6115778; doi:10.3390/ani8080142)
Supplement: Supplementary file 1 [file animals-08-00142-s001.pdf]

a.

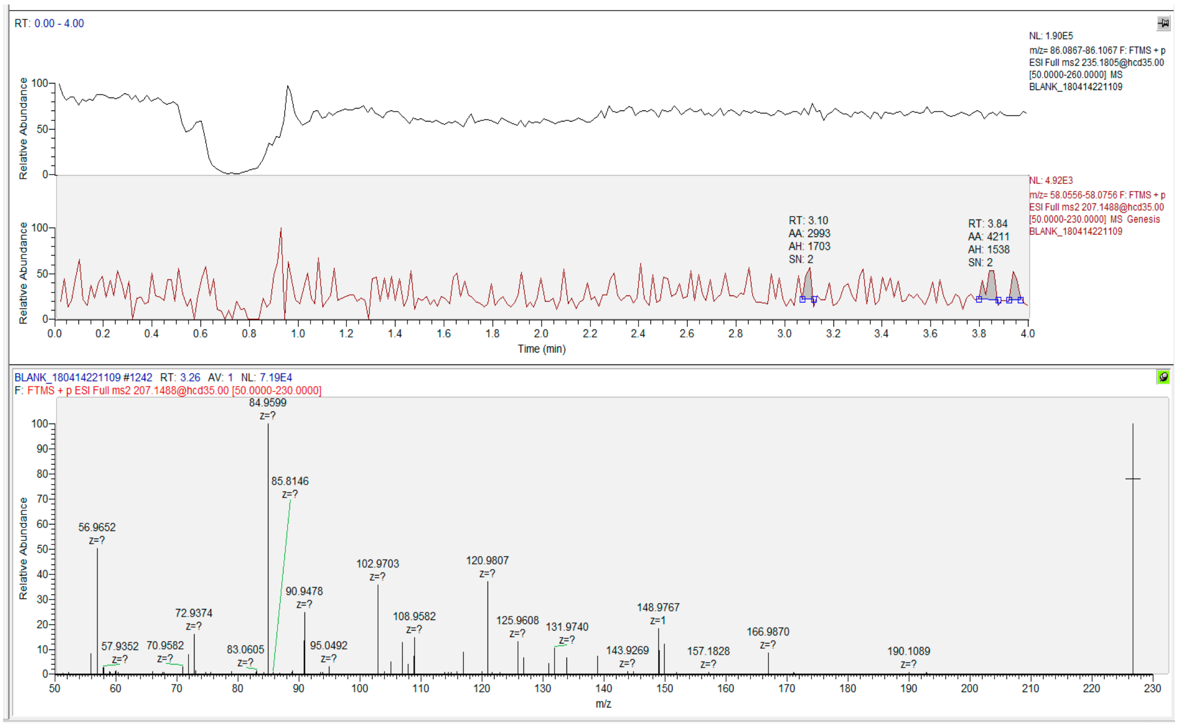

RT: 0.00 - 4.00
NL: 1.90E5
m/z= 86.0867-86.1067 F: FTMS + p ESI Full ms2 235.1805@hcd35.00 [50.0000-260.0000] MS BLANK_180414221109
NL: 4.92E3
m/z= 58.0556-58.0756 F: FTMS + p ESI Full ms2 207.1488@hcd35.00 [50.0000-230.0000] MS Genesis BLANK_180414221109
RT: 3.10 AA: 2993 AH: 1703 SN: 2
RT: 3.84 AA: 4211 AH: 1538 SN: 2
Relative Abundance
Time (min)
BLANK_180414221109 #1242 RT: 3.26 AV: 1 NL: 7.19E4
F: FTMS + p ESI Full ms2 207.1488@hcd35.00 [50.0000-230.0000]
84.9599 z=?
85.8146 z=?
56.9652 z=?
102.9703 z=?
120.9807 z=?
90.9478 z=?
148.9767 z=1
72.9374 z=?
108.9582 z=?
125.9608 z=?
131.9740 z=?
166.9870 z=?
57.9352 z=?
70.9582 z=?
83.0605 z=?
95.0492 z=?
143.9269 z=?
157.1828 z=?
190.1089 z=?
m/z

b.

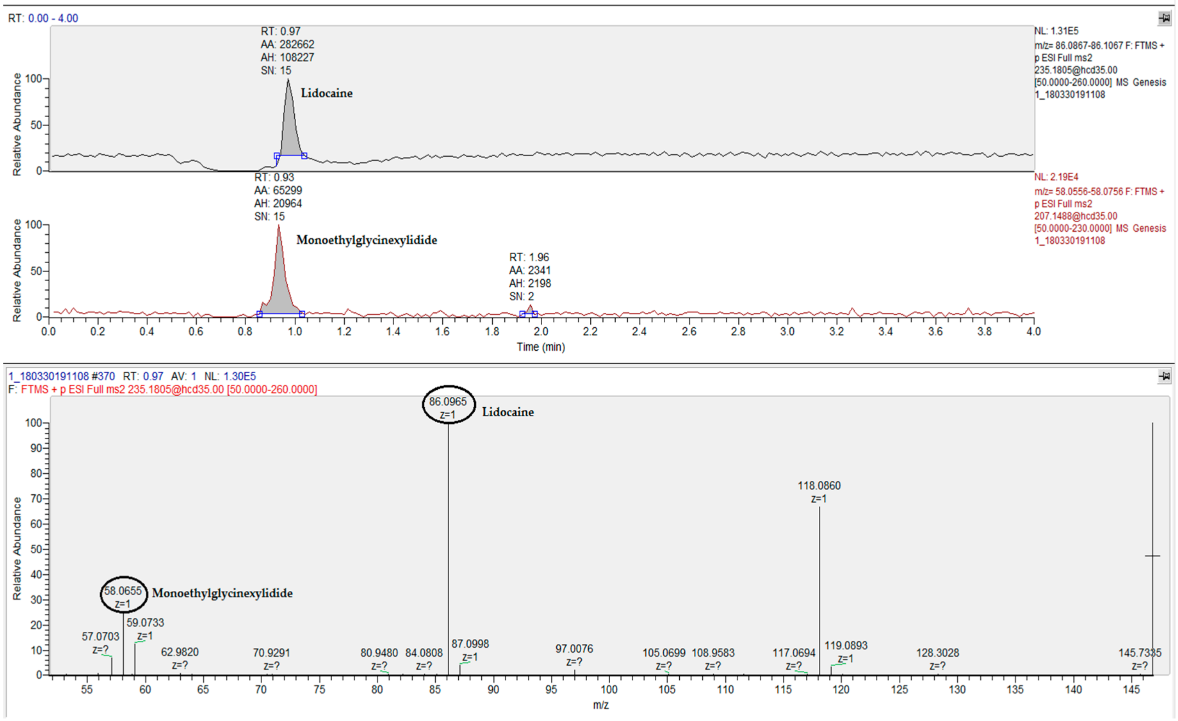

RT: 0.00 - 4.00
RT: 0.97 AA: 282662 AH: 108227 SN: 15
Lidocaine
NL: 1.31E5
m/z= 86.0867-86.1067 F: FTMS + p ESI Full ms2 235.1805@hcd35.00 [50.0000-260.0000] MS Genesis 1_180330191108
RT: 0.93 AA: 65299 AH: 20964 SN: 15
Monoethylglycinexylidide
RT: 1.96 AA: 2341 AH: 2198 SN: 2
NL: 2.19E4
m/z= 58.0556-58.0756 F: FTMS + p ESI Full ms2 207.1488@hcd35.00 [50.0000-230.0000] MS Genesis 1_180330191108
Relative Abundance
Time (min)
1_180330191108 #370 RT: 0.97 AV: 1 NL: 1.30E5
F: FTMS + p ESI Full ms2 235.1805@hcd35.00 [50.0000-260.0000]
86.0965 z=1
Lidocaine
118.0860 z=1
58.0655 z=1
Monoethylglycinexylidide
59.0733 z=1
57.0703 z=?
62.9820 z=?
70.9291 z=?
80.9480 z=?
84.0808 z=?
87.0998 z=1
97.0076 z=?
105.0699 z=?
108.9583 z=?
117.0694 z=?
119.0893 z=1
128.3028 z=?
145.7335 z=?
m/z

c.

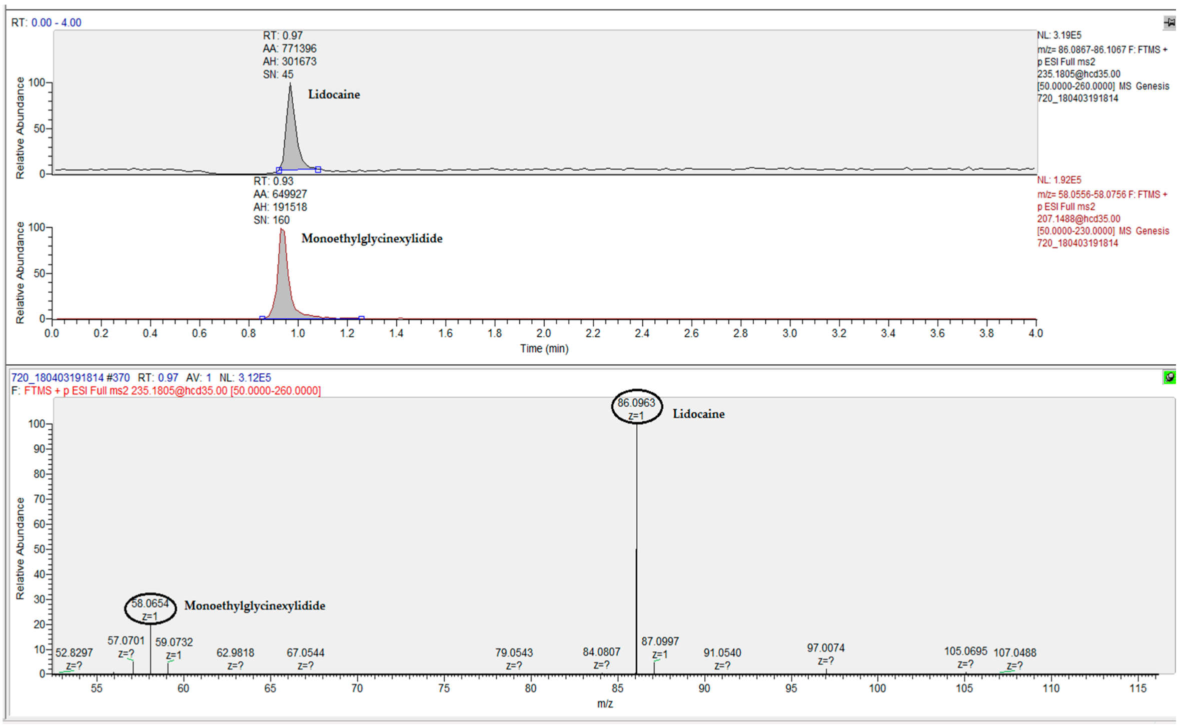

Supplementary Figure S1. Chromatograms and mass spectra (obtained in PRM mode) of (**a**) blank goat plasma, (**b**) blank plasma spiked with 0.00125 µg/mL of lidocaine and monoethylglycinexylidide, and (**c**) plasma from an experimental animal after cornual nerve block.

a.

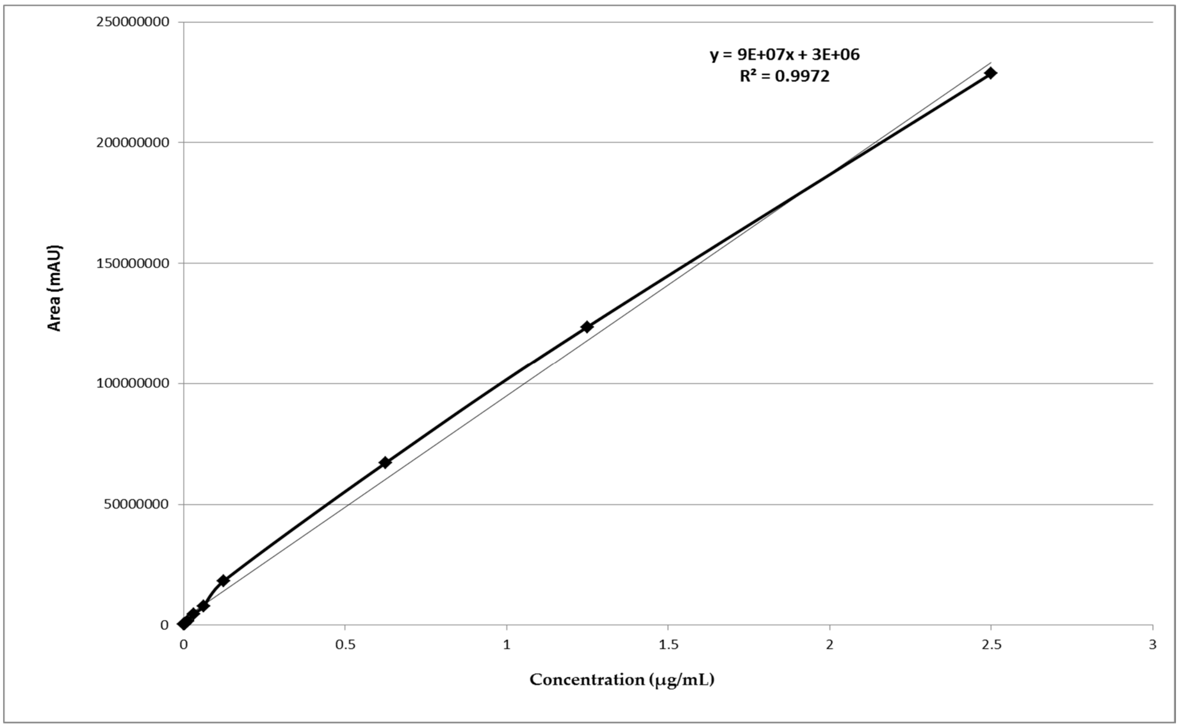

b.

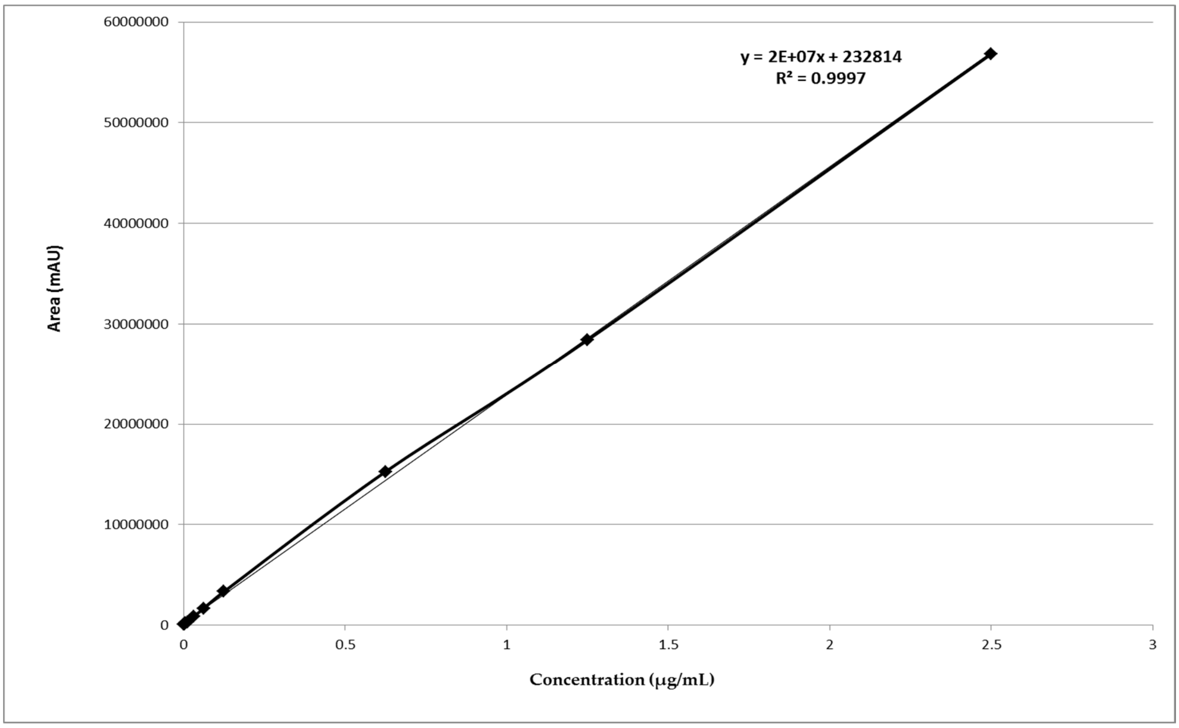

Supplementary Figure S2. Calibration curves (0.00125 – 2.50 µg/mL) of lidocaine (**a**) and monoethylglycinexylidide; (**b**) constructed by spiking pooled plasma from untreated goat kids.
